# Clarification of the Position of *Linum stelleroides* Planch. within the Phylogeny of the Genus *Linum* L.

**DOI:** 10.3390/plants11050652

**Published:** 2022-02-27

**Authors:** Nadezhda L. Bolsheva, Nataliya V. Melnikova, Ekaterina M. Dvorianinova, Liudmila N. Mironova, Olga Y. Yurkevich, Alexandra V. Amosova, George S. Krasnov, Alexey A. Dmitriev, Olga V. Muravenko

**Affiliations:** 1 Engelhardt Institute of Molecular Biology, Russian Academy of Sciences, 119991 Moscow, Russia; mnv-4529264@yandex.ru (N.V.M.); dvorianinova.em@phystech.edu (E.M.D.); olikys@gmail.com (O.Y.Y.); amomar@mail.ru (A.V.A.); gskrasnov@mail.ru (G.S.K.); olgmur1@yandex.ru (O.V.M.); 2 Moscow Institute of Physics and Technology, 141701 Moscow, Russia; 3 Botanical Garden-Institute of the Far Eastern Branch of the Russian Academy of Sciences, 690024 Vladivostok, Russia; lymironova@yandex.ru

**Keywords:** flax, *Linum stelleroides* Planch., phylogeny, genome sequencing

## Abstract

The phylogeny of members of the family *Linaceae* DC. ex Perleb has not been adequately studied. In particular, data on the phylogenetic relationship between *Linum stelleroides* Planch. and other representatives of the blue-flowered flax are very controversial. In the present work, to clarify this issue, we obtained DNA sequences of three nuclear loci (IGS and ITS1 + 5.8S rDNA + ITS2 of the 35S rRNA gene and the 5S rRNA gene) and eight chloroplast loci (*rbcL*, the *trnL*–*trnF* intergenic spacer, *matK*, the 3′ *trnK* intron, *ndhF*, *trnG*, the *psbA*–*trnH* intergenic spacer, and *rpl16*) of 10 *Linum* L. species (*L. stelleroides*, *L. hirsutum*, *L. perenne*, *L. leonii*, *L. lewisii*, *L. narbonense*, *L. decumbens*, *L. grandiflorum*, *L. bienne* (syn. *L. angustifolium*), and *L. usitatissimum*) using high-throughput sequencing data. The phylogenetic analysis showed that *L. stelleroides* forms a basal branch in the blue-flowered flax clade. Previously found inconsistencies in the position of *L. stelleroides* and some other species in the *Linaceae* phylogenetic tree resulted from the erroneous species identification of some of the studied plant samples.

## 1. Introduction

*Linum stelleroides* Planch., an annual, wild-growing species, was named after German naturalist Georg Steller, who explored the nature of Siberia, the Far East, and Alaska. The species is distributed across the southern Far East and Eastern Siberia in Russia, China, Japan, Korea, Kyrgyzstan, Tajikistan, Turkmenistan, and Uzbekistan. Based on several morphological characteristics, *L. stelleroides* differs significantly from other members of the genus *Linum*. Notably, in *L. stelleroides*, the margins of the sepals are membranous, with large black stipulate glands. *L. stelleroides* has pantoporate pollen grains with 12 pores, whereas the members of the other three sections have tricolpate pollen, except for a few polyploid species which have polycolpate pollen [1,2]. These differences provided the basis for categorizing *L. stelleroides* within a separate monotypic section of *Stellerolinum* Juz. [1,2,3,4].

Recent molecular phylogenetic studies showed that representatives of the genus *Linum* are subdivided into two large sister groups. The first group comprises yellow-flowered species corresponding to sections *Cathartolinum* (Reichen b.) Griseb., *Syllinum* Griseb., and *Linopsis* (Reichenb.) Engelmann and genera *Cliococca* Bab., *Hesperolinon* (A. Gray) Small, *Radiola* Hill., and *Sclerolinon* C.M. Rogers. The second group includes blue-flowered flax species from sections *Dasylinum* (Planchon) Juz., *Adenolinum* (Reichenb.) Juz. (syn. *L. perenne* L. group), and *Linum* L. and *L. stelleroides* (monotypic sect. *Stellerolinum* Juz. ex Prob.) [5].

At the same time, data on the phylogenetic relationship between *L. stelleroides* and other representatives of the blue-flowered flax are very contradictory. In particular, several molecular phylogenetic studies indicated that *L. stelleroides* is closely related to representatives of the sect. *Adenolinum* (syn. *L. perenne* group) [6,7,8,9], whereas, in the *rbcL* phylogeny, it was a sister to the sect. *Dasylinum* [5]. According to a sequence-specific amplified polymorphism (SSAP) analysis and data on nuclear internal transcribed spacers (ITS), three chloroplast DNA sequences (*ndhF* + *trnK* intron + *trnL*-*trnF* spacer), and the nuclear 5S ribosomal RNA (rRNA) gene, *L. stelleroides* formed an independent basal clade of the blue-flowered flax [5,10,11]. Finally, combined data on ITS and seven plastid sequences (*rbcL*, the *trnL*–*trnF* spacer, the 3′ *trnK* intron, *ndhF*, *trnG*, the *psbA*–*trnH* spacer, the *rpl16* intron) showed that *L. stelleroides* takes an intermediate position between the clusters formed by representatives of the sect. *Dasylinum* on the one hand, and representatives of the sect. *Adenolinum* on the other hand [12].

In this study, we used recent high-throughput sequencing results for several *Linum* species, including *L. stelleroides*, to determine or improve the accuracy of the DNA sequences of three nuclear loci (IGS and ITS1 + 5.8S rDNA + ITS2 sequences of the 35S rRNA gene and the 5S rRNA gene) and eight chloroplast loci (*rbcL*, the *trnL*–*trnF* intergenic spacer, *matK*, the 3′ *trnK* intron, *ndhF*, *trnG*, the *psbA*–*trnH* intergenic spacer, *rpl16*). The improved sequences were used to clarify the phylogenetic relationship between *L. stelleroides* and other representatives of the genus *Linum*.

## 2. Results

### 2.1. Morphological Characteristics of L. stelleroides Specimens

**Figure 1:**
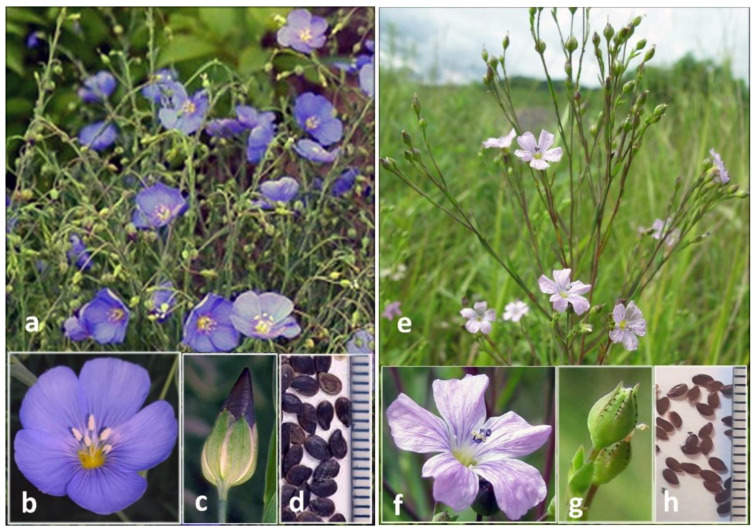
Photo of plants of the LIN 1655 accession (**a**–**d**) and *L. stelleroides* specimen from the Far East of Russia (**e**–**h**). **a**,**e**—general view of plants; **b**,**f**—flower morphology; **c**,**g**—calyx morphology; **d**,**h**—seeds, scale 1 mm. Photo of *L. stelleroides* in its natural habitat near the Kraskino village kindly provided by Dr. Z.V. Kozhevnikova.

To study the morphology of mature plants, seeds of the *L. stelleroides* accession LIN 1655 from the Germplasm Bank of the Leibniz Institute of Plant Genetics and Crop Plant Research, Germany (IPK) were planted in the field and grown to the stages of flowering and seed maturation. The grown LIN 1655 plants were morphologically different from *L. stelleroides*. Namely, LIN 1655 is perennial; its flowers are heterostylous; the petals are blue-violet; the outer sepals are 4–6 mm with a sharp apex and a narrow white scarious margin; the inner sepals are wider and slightly longer than the outer ones with a rounded apex and entire scarious margins; capsules are 5–6 mm long and 4–5 mm wide, subglobose, with a very short beak; and the seeds are quite large, i.e., 3.0–3.5 mm in length and dark brown in color (Figure 1a–d). According to botanical descriptions [3,4], *L. stelleroides* is an annual; its flowers are ~1 cm in diameter; its petals are pink-violet, sometimes blue-violet, and rarely white; the sepals are 3–5 mm long and rounded ovate with a sharp apex; the sepal margins are narrowly bordered with characteristic sessile rounded black glands of a rather large size; capsules are acuminate, subglobose, 4–5 mm long and 4–5 mm wide; and the seeds are rather small, i.e., ~2 mm in length and brown colored.

Thus, in terms of morphological characteristics, the LIN 1655 accession is similar to species of the *L. perenne* group, but not *L. stelleroides*. Moreover, the morphological features of the *L. stelleroides* specimen collected in the Far East of Russia correspond completely to those described at the end of the previous paragraph (Figure 1e–h).

### 2.2. Assembling Sequences

At the initial stage of our work, sequences of 11 loci (IGS and ITS1 + 5.8S rDNA + ITS2 of the 35S rRNA gene, the 5S rRNA gene, *rbcL*, the *trnL*–*trnF* intergenic spacer, *matK*, the 3′ *trnK* intron, *ndhF*, *trnG*, the *psbA*–*trnH* intergenic spacer, and *rpl16*) were assembled from whole-genome sequencing (WGS) data for 12 flax species: *L. stelleroides*, *L. hirsutum*, *L. perenne*, *L. narbonense*, and *L. usitatissimum* (NCBI BioProject PRJNA497747, our data) and *L. leonii*, *L. lewisii*, *L. decumbens*, *L. grandiflorum*, *L. bienne* (syn. *L. angustifolium*), *L. strictum*, and *L. arboreum* (PRJNA262458). The resulting sequences were compared with the corresponding ones from NCBI GenBank, previously obtained by sequencing of cloned PCR amplicons in several studies. All the assembled sequences for *L. usitatissimum*, *L. bienne*, *L. decumbens*, *L. narbonense*, *L. perenne*, *L. leonii*, *L. lewisii*, and *L. hirsutum* turned out to be highly homologous (homology level >98%) to the sequences of the same or closely related species from GenBank. At the same time, a small number of single nucleotide substitutions, deletions, or insertions in polymorphic loci were observed.

For the *L. stelleroides* and *L. grandiflorum* species, the assembled *rbcL*, *trnL*–*trnF* intergenic spacer, 3’ *trnK* intron, *ndhF*, and ITS sequences were highly homologous to the ones listed in GenBank for the same species (homology level ~99%). However, the homology between the corresponding sequences of *trnG*, the *psbA*–*trnH* intergenic spacer, and *rpl16* was significantly lower (<90%). At the same time, *trnG*, the *psbA*–*trnH* intergenic spacer, and *rpl16* sequences presented in GenBank for *L. stelleroides* and *L. grandiflorum* appeared to be highly homologous (>99%) to the sequences of the same loci for members of the sect. *Adenolinum*. Notably, for the sequences of *rbcL*, the *trnL*–*trnF* intergenic spacer, the 3’ *trnK* intron, *ndhF*, and ITS, such a high homology between *L. stelleroides* or *L. grandiflorum* and sect. *Adenolinum* members was not observed, suggesting that a mistake in the identification of species of plant specimens was made in the course of earlier sequencing of *trnG*, the *psbA*–*trnH* intergenic spacer, and *rpl16* of *L. stelleroides* and *L. grandiflorum*.

For all the analyzed loci of *L. strictum* (sect. *Linopsis*) and *L. arboreum* (sect. *Syllinum*), we revealed a low level of homology between the sequences presented in GenBank and those assembled from WGS reads in this study. At the same time, the assembled *L. strictum* sequences were highly homologous (>99%) to those of the corresponding loci of sect. *Adenolinum* members deposited in GenBank. The sequences of chloroplast loci assembled from genomic reads of *L. arboreum* showed low homology to the GenBank sequences of both this and other species of the family *Linaceae*. We could not assemble the ITS1 + 5.8S rDNA + ITS2 fragment of the 35S rRNA gene for *L. arboreum* using the reference from GenBank. Therefore, we had to use the Repeat Explorer 2 toolkit and obtained the fragment showing <40% homology to similar sequences of members of the genus *Linum* and significant homology (99%) to the ITS1 + 5.8S rDNA + ITS2 sequence of *Alyssoides utriculata* (L.) Medikus (*Brassicaceae*), accession KF022514 in NCBI GenBank. These results gave strong reasons to doubt the correctness of the species identification of the sequenced *L. strictum* and *L. arboreum* accessions. Therefore, the sequences of these two species assembled from WGS reads were excluded from further phylogenetic analysis. The results of assembling the sequences of the studied loci for the remaining 10 flax species are presented in Supplementary Tables S1–S11.

### 2.3. Phylogenetic Analysis

**Figure 2:**
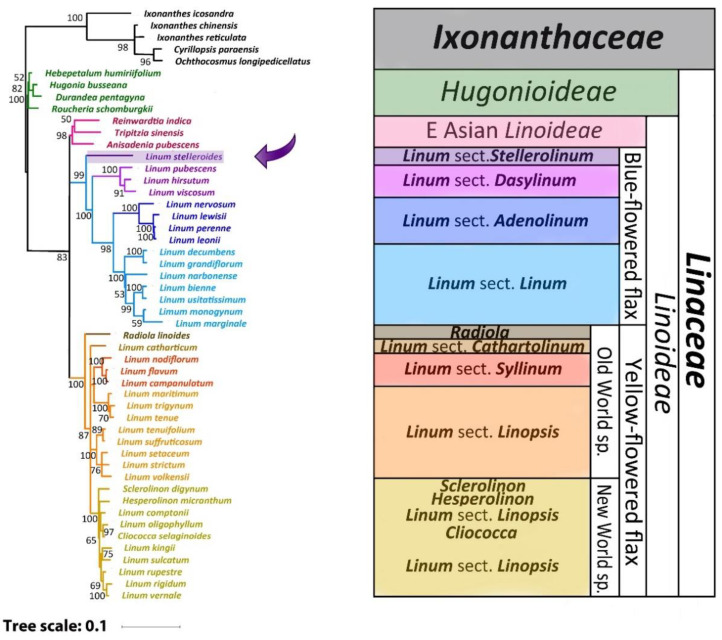
Maximum likelihood (ML) phylogeny of 11 loci of 45 *Linaceae* taxa and the selected outgroup of 5 *Ixonanthaceae* taxa. The values shown above or below branches are the node supports assessed by bootstrap values. The position of *L. stelleroides* is marked with an arrow.

**Figure 3:**
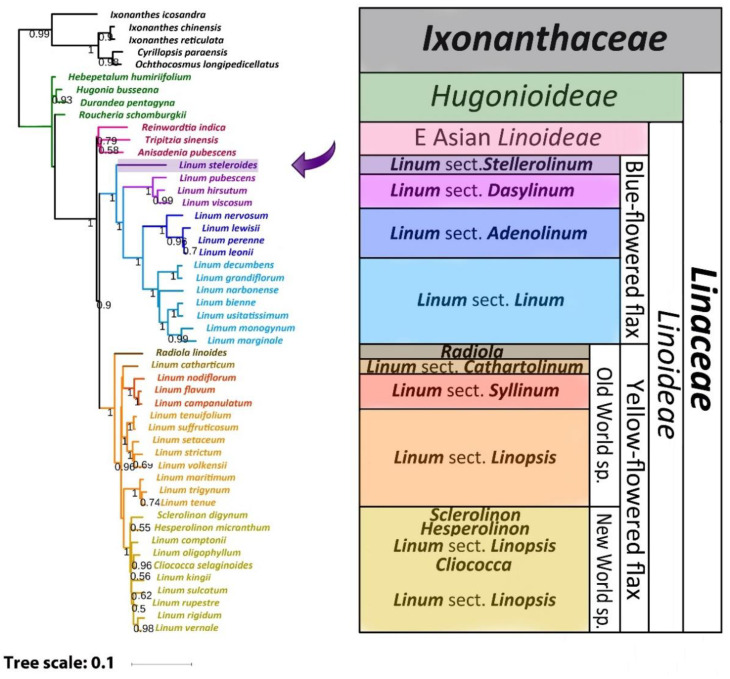
Bayesian phylogeny of 11 loci of 45 *Linaceae* taxa and the selected outgroup of 5 *Ixonanthaceae* taxa. The values shown above or below branches are the node supports assessed by the Bayesian-like modification of the approximate likelihood ratio test (aBayes). The position of *L. stelleroides* is marked with an arrow.

**Figure 4:**
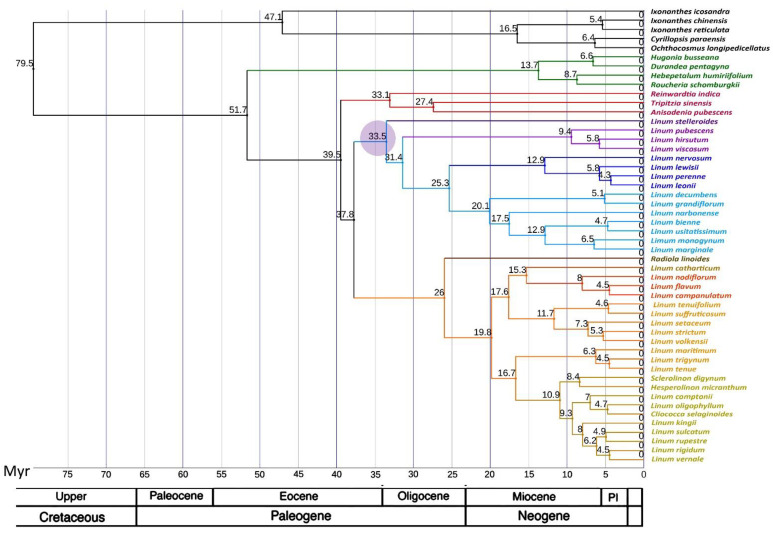
Bayesian chronogram of 45 taxa of *Linaceae* and 5 taxa of *Ixonanthaceae*. The values shown above branches are the dates of the nodes. The purple circle marks the point of the sect. *Stellerolinum* divergence from the common trunk of the blue-flowered flax. Pl on the geochronological scale—Pliocene.

Based on GenBank data for the sequences of 11 loci for 40 plant species and the updated sequences of the same loci assembled from WGS reads for the other 10 species of flax, Maximum Likelihood (ML) and Bayesian phylogenetic trees of *Linoideae* were constructed (Figure 2 and Figure 3). The performed phylogenetic analysis showed that *L. stelleroides* represents an independent branch in the phylogenetic tree of *Linaceae*, occupying the basal position inside the clade of the blue-flowered flax. The divergence of the *L. stelleroides* ancestor from the common trunk of the blue-flowered flax clade occurred approximately 33.5 million years ago (Figure 4). Then, species of sections *Dasylinum*, *Adenolinum*, and *Linum* branched off from their common trunk. The basal branch of the yellow-flowered flax clade is represented by *Radiola linoides* Roth. Then, branches of species of sections *Cathartolinum*, *Syllinum*, and *Linopsis* diverged from the basal one. The sect. *Linopsis* split into three clades—one of them is represented by species of the New World, and the other two— species of the Old World with the main chromosome number equal to 9 (*L. setaceum*, *L. strictum*, *L. volkensii*, *L. suffruticosum*, *L. tenuifolium*) and 10 (*L. maritimum*, *L. trigynum*, *L. tenue*).

## 3. Discussion

### 3.1. The Reasons for Some Inconsistencies in Previous Phylogenetic Studies

The differences in the location of *L. stelleroides* in *Linaceae* phylogenetic trees constructed by different authors prompted us to examine the reasons for these discrepancies in detail. In previous studies, when the seeds of the LIN 1655 accession (IPK) or daughter accessions—CN 107274 (Plant Gene Resources of Canada, PGRC) and PI 650330 (Western Regional Plant Introduction Station, USDA-ARS NPGS, Pullman, WA, USA)—were used as a source of *L. stelleroides* genomic DNA, analyses demonstrated an extremely high similarity of the examined loci sequences of *L. stelleroides* to those of sect. *Adenolinum* members. According to our previous study [13], the karyotype of LIN 1655 contains 18 chromosomes and cannot be distinguished from that of sect. *Adenolinum* members. At the same time, Sokolovskaya and Probatova, who studied *L. stelleroides* collected in its natural habitat in the Russian Far East, showed that the karyotype of this species contains 20 chromosomes [14]. Having been grown in soil, the plants of the LIN 1655 accession differed significantly from *L. stelleroides* in a number of morphological characteristics and looked like representatives of the *L. perenne* group (sect. *Adenolinum*). Therefore, this sample (or the ones derived from it) was clustered together with representatives of the *L. perenne* group in molecular phylogenetic studies [6,7,8,9]. In contrast, *L. stelleroides* was located in a phylogenetic tree separately from the species of the sect. *Adenolinum* in phylogenetic studies based on the use of herbarium plant samples as a source of genomic DNA [5], enabling the control of the species identification correctness. In another study [12], to construct a phylogeny, the sequences of *rbcL*, the *trnL*–*trnF* spacer, the 3’ *trnK* intron, *ndhF*, and ITS of *L. stelleroides* herbarium samples [5] were combined with the sequences of *trnG*, the *psbA*–*trnH* spacer, and the *rpl16* intron obtained from genomic DNA of the CN 107274 accession (PGRC), which was derived from LIN 1655. Therefore, *L. stelleroides* took an intermediate position between the representatives of sections *Linum* and *Adenolinum* in a phylogenetic tree.

In this work, as well as in our previous studies [10,11], we used a sample of *L. stelleroides* collected in the natural habitat in the Far East of Russia and grown in the Botanical Garden-Institute of the Far-Eastern Branch of the Russian Academy of Sciences (BGI FEB RAS) in Vladivostok, Russia. It has all the specific morphological characteristics inherent in the *L. stelleroides* species. Previously, we studied this sample using molecular karyological methods and found that its chromosome number is 2n = 20 [10], which completely agrees with the results of Sokolovskaya and Probatova [14]. In addition, the sequences of *rbcL*, the *trnL*–*trnF* spacer, *ndhF*, and ITS assembled from WGS reads of this specimen turned out to be highly homologous to the sequences of the same loci in the genomic DNA of herbarium samples [5].

Along with morphological and karyological similarities [3,10], closely related *L. decumbens* and *L. grandiflorum* showed a high degree of similarity between their *rbcL*, *trnL*–*trnF* spacer, 3’ *trnK* intron, *ndhF*, and ITS sequences [5]. The sequences of these loci assembled in this study proved to be highly homologous (>99%) to those obtained for herbarium samples [5]. At the same time, the sequences of *trnG*, the *psbA*–*trnH* spacer, and the *rpl16* intron of *L. grandiflorum* (NCBI GenBank accessions GQ845264, GQ845291, and GQ845237 respectively) obtained from genomic DNA of the Tmp1200 seed accession (PGRC) [12] were more similar to the sequences of the same loci of the *L. perenne* group species than to those of *L. decumbens* and *L. grandiflorum* assembled from WGS reads. Thus, a seed sample of *L. grandiflorum* was likely confused with that of one of sect. *Adenolinum* members. Having assembled the examined loci from WGS reads of two yellow-flowered flax species —*L. strictum* and *L. arboreum*, we also found errors in the species identification of the plant samples. Thus, the WGS data for the specimen named *L. strictum* can belong to a member of the sect. *Adenolinum*. In the phylogenetic tree, this WGS data were clustered together with that of sect. *Adenolinum* members [9]. The WGS data for *L. arboreum* probably belongs to *Alyssoides utriculata* (L.) Medik. (*Brassicaceae*) or to a closely related species.

Thus, the analysis of both our data and the literature demonstrated that seed collections contain a significant number of samples with erroneous species identification. The correctness of the species identification of a studied plant specimen has to be re-examined before starting molecular genetic studies.

### 3.2. Comparison of Our Results with Previous Phylogenetic Studies of Flax

The obtained ML and Bayesian trees in an initial nonclock analysis of the combined extant data set (Figure 2 and Figure 3) are largely in agreement with previous phylogenetic studies. At the same time, in the published phylogenies, we observed differences in the arrangement of *L. stelleroides* and *L. grandiflorum* inside the clade of the blue-flowered flax [5,6,7,8,9,10,11,12]. These differences are associated with erroneous species identification of the used *L. stelleroides* and *L. grandiflorum* samples. After eliminating these errors, the phylogenetic relationship between these species was clarified. *L. stelleroides* forms a basal branch of the blue-flowered flax clade, and *L. grandiflorum* is closely related to *L. decumbens*. The phylogenetic relationship in the clade of yellow-flowered flax species established in this study is also in good agreement with the previously obtained data. However, the phylogenetic relationship between sections *Cathartolinum* and *Syllinum*, as well as the members of the sect. *Linopsis*, is still not entirely clear. Since the data on genomic sequences of representatives of the yellow-flowered flax clade are currently highly limited, their genomes require further research using modern sequencing methods.

## 4. Materials and Methods

### 4.1. Data Sources

To clarify the position of *L. stelleroides* in the phylogenetic tree of *Linaceae*, eight regions of chloroplast DNA (*matK*, *ndhF*, *rbcL*, the *rpl16* intron, *trnG*, the *psbA*–*trnH* intergenic spacer, the 3’ *trnK* intron, and the *trnL*–*trnF* intergenic spacer) and three nuclear sequences (IGS and ITS1 + 5.8S rDNA + ITS2 of the 35S rRNA gene and the 5S rRNA gene) were analyzed in 50 plant species, representing an almost complete generic and sectional sampling of *Linoideae.* Four *Hugonioideae* species and five *Ixonanthaceae* species were taken as an external group. Examined plant species and the sources of the analyzed sequences are listed in Supplementary Table S12.

For the majority of the studied species, we used sequences from NCBI GenBank, obtained by PCR amplification followed by amplicon cloning and sequencing. Also, we took into the analysis sequences of 5S rRNA genes of the blue-flowered flax, which were previously determined using PCR amplification and subsequent high-throughput sequencing of amplicons [10]. Shotgun genomic sequencing data for 12 flax species (*L. stelleroides*, *L. hirsutum*, *L. perenne*, *L. leonii*, *L. lewisii*, *L. narbonense*, *L. decumbens*, *L. grandiflorum*, *L. bienne* (syn. *L. angustifolium*), *L. usitatissimum*, *L. strictum*, and *L. arboreum*) were derived earlier and deposited in NCBI SRA (BioProjects PRJNA262458 and PRJNA497747). Five flax samples representing *L. stelleroides*, *L. hirsutum*, *L. perenne*, *L. narbonense*, and *L. usitatissimum* species were sequenced within our BioProject PRJNA497747. In 2012, a number of accessions were grown in the field to verify the correctness of species identification, including *L. hirsutum*, *L. perenne*, and *L. narbonense* (each of the three was sequenced later), as well as LIN 1655 from Leibniz Institute of Plant Genetics and Crop Plant Research (Gatersleben, Germany) [11]. Considering the results of this test, we excluded the LIN 1655 accession from further studies and replaced it with the *L. stelleroides* specimen, the seeds of which were collected near the Kraskino village in Khasansky district of the Far East of Russia (natural habitat) and grown in the Botanical Garden-Institute FEB RAS, Vladivostok, Russia. As LIN 1655 continues to be actively used in various genomic studies, we decided to present here previously unpublished information regarding the morphology of this sample. For all 12 sequenced species, we performed a local reference-based assembly of orthologous loci suitable for further phylogenetic study using the CLC Genomics Workbench software (CLC bio, Aarhus, Denmark). We used the corresponding gene sequence of the same or closely related species obtained by cloning and sequencing of PCR products and deposited in GenBank as a reference for each of the studied loci. When creating consensus sequences for loci containing a SNP, the most common nucleotide variant was chosen. Intergenic spacer (IGS) sequences of the flax 35S rRNA genes were assembled with the TAREAN tool of RepeatExplorer2 [15] using the option “cluster merging”.

### 4.2. Phylogenetic Analysis and Statistical Evaluation

The alignment of the analyzed sequences was performed using ClustalW [16] with the following parameters for Pairwise Sequence Alignment: Gap Opening Penalty—15, Gap Extension Penalty—6.66. The parameters for Multiple Sequence Alignment were: Gap Opening Penalty—15, Gap Extension Penalty—6.66, DNA Weight Matrix—JUB, Transition Weight—0.5, Delay Divergent Cutoff—70%.

A phylogenetic analysis was performed using the Maximum Likelihood (ML) method with MEGA version X [17] and Bayesian analysis with mrBayes [18,19]. For both phylogenetic methods, the best fit model of nucleotide substitution was determined by the JModelTest 2.1.1.0 software [20]. The selected model was GTR + I + G (the general time-reversible model with a proportion of invariable sites and a gamma-shaped distribution of rates across sites) established by both corrected Akaike Information Criterion (AICc) and Bayesian Information Criterion (BIC). In the ML analysis, support for branches was evaluated using the nonparametric bootstrap (BS) with 500 replicates.

A Bayesian analysis was performed using the GTR + I + G model. Prior probability distributions for all parameters were kept at default values, and all the parameters were unlinked between all partitions except for tree topology and branch lengths. MrBayes was run with Markov chains Monte Carlo (MCMC) analysis of four (one cold and three heated) chains, two runs, 4 million generations, sampling one tree in every 1000 generations. Node supports were assessed by the Bayesian-like modification of the approximate likelihood ratio test (aBayes) [21]. The Bayesian analysis was repeated twice.

Divergence times of *Linaceae* taxa were estimated using an uncorrelated log-normal relaxed clock model [19]. The general time-reversible model with gamma rate variation across sites (GTR + G) was preferred for all partitions. The time tree was calibrated by two points using total-evidence dating. The oldest known fossil pollen grains of *Linum* dated to 33.9–37.2 Ma [22]. The age of this fossil was calibrated as uniformly distributed inside the interval (33.9–37.2 Ma). The calibration of the tree age was based on the recent estimation of divergence time of *Linaceae* and *Ixonanthaceae:* 103.4–73.6 Ma [23]. To date the root of the tree, we used an offset exponential distribution with minimal age equal to 70 Ma and mean age equal to 90 Ma. The MCMC analysis of four chains with two independent runs was carried out until the average standard deviation of split frequencies became <0.01. The ML and Bayesian trees were visualized by the iTOL online tool (https://itol.embl.de/, accessed on 10 January 2022) [24].

## Data Availability

The data presented in this study is contained within the article and Supplementary Materials.

## Supplementary Materials

The following supporting information can be downloaded at: https://www.mdpi.com/article/10.3390/plants11050652/s1, Table S1: Assembled sequences of IGS of the 35S rRNA gene; Table S2: Assembled sequences of ITS1 + 5.8S rDNA + ITS2 of the 35S rRNA gene; Table S3: Assembled sequences of the 5S rRNA gene; Table S4: Assembled sequences of *matK*; Table S5: Assembled sequences of *ndhF*; Table S6: Assembled sequences of *rbcL*; Table S7: Assembled sequences of the *rpl16* intron; Table S8: Assembled sequences of *trnG*; Table S9: Assembled sequences of the *psbA*–*trnH* intergenic spacer; Table S10: Assembled sequences of the 3’ *trnK* intron; Table S11: Assembled sequences of the *trnL*–*trnF* intergenic spacer; Table S12: Sources of flax sequences used for the phylogenetic study: NCBI GenBank accessions and supplementary tables containing sequences assembled by us using high-throughput sequencing data.
