# Supplementary material for: Clarification of the Position of Linum stelleroides Planch. within the Phylogeny of the Genus Linum L
Source: Plants (Basel). 2022 Feb 27;11(5):652. doi: 10.3390/plants11050652 (PMC8912650; doi:10.3390/plants11050652)
Supplement: Supplementary file 1 [file plants-11-00652-s001.zip › Supplementary Table S12.pdf]

Table S12. Sources of flax sequences used for the phylogenetic study: NCBI GenBank accessions and supplementary tables containing sequences assembled by us using high-throughput sequencing data.

Species	IGS	ITS	5S rDNA	*matK*	*ndhF*	*rbcL*	*rpl16*	*trnG*	*psbA–trnH*	*trnK*	*trnL–trnF*
Ixonanthes chinensis						AY788179					
Ixonanthes icosandra						AF206783					KU853222
Ixonanthes reticulata				AB233789		AB233893					
Cyrillopsis paraensis				FJ670024	FJ670100	FJ038065					FJ039280
Ochthocosmus longipedicellatus					FJ670101	FJ707535				EF135573	
Hugonia busseana		FJ169512		HM544087	FJ160773	HM544052				FJ160817	FJ160857
Durandea pentagyna				HM544081		FJ169559				FJ670027	
Hebepetalum humiriifolium				HM544082		HM544047			MF786115		
Roucheria schomburgkii				HM544121		FJ169603					
Reinwardtia indica		FJ169514		AB048380	FJ160814	FJ169599				FJ160853	FJ160898
Tirpitzia sinensis		FJ169515		HM544124	FJ160816	FJ169602				FJ160855	FJ160900
Anisadenia pubescens		FJ169513		HM544078	FJ160772	FJ169557					FJ160856
Linum stelleroides	Table S1	Table S2	Table S3	Table S4	Table S5	Table S6	Table S7	Table S8	Table S9	Table S10	Table S11
Linum hirsutum	Table S1	Table S2	Table S3	Table S4	Table S5	Table S6	Table S7	Table S8	Table S9	Table S10	Table S11
Linum viscosum		FJ169517			FJ160791	FJ169584				FJ160830	FJ160875
Linum pubescens		KU674812			FJ160790	FJ169585				FJ160829	FJ160874

Linum nervosum		MH592583		HM544110		HM544071					
Linum perenne	Table S1	Table S2	Table S3	Table S4	Table S5	Table S6	Table S7	Table S8	Table S9	Table S10	Table S11
Linum leonii	Table S1	Table S2	Table S3	Table S4	Table S5	Table S6	Table S7	Table S8	Table S9	Table S10	Table S11
Linum lewisii	Table S1	Table S2	Table S3	Table S4	Table S5	Table S6	Table S7	Table S8	Table S9	Table S10	Table S11
Linum narbonense	Table S1	Table S2	Table S3	Table S4	Table S5	Table S6	Table S7	Table S8	Table S9	Table S10	Table S11
Linum decumbens	Table S1	Table S2	Table S3	Table S4	Table S5	Table S6	Table S7	Table S8	Table S9	Table S10	Table S11
Linum grandiflorum	Table S1	Table S2	Table S3	Table S4	Table S5	Table S6	Table S7	Table S8	Table S9	Table S10	Table S11
Linum bienne	Table S1	Table S2	Table S3	Table S4	Table S5	Table S6	Table S7	Table S8	Table S9	Table S10	Table S11
Linum usitatissimum	Table S1	Table S2	Table S3	Table S4	Table S5	Table S6	Table S7	Table S8	Table S9	Table S10	Table S11
Linum monogynum				HM544108		HM544069					
Linum marginale		FJ169528	Table S3		FJ160804					FJ160843	FJ160888
Radiola linoides		FJ169534		HM544118	FJ160815	FJ169598				FJ160854	FJ160899
Linum catharticum		FJ169533		HM544103	FJ160796	FJ169570				FJ160835	FJ160880
Linum nodiflorum		KU674810	Table S3		FJ160795	FJ169581	GQ845232	GQ845259	GQ845286	FJ160834	FJ160879
Linum flavum		FJ169538		HM544105	FJ160794	FJ169574	GQ845228	GQ845255	GQ845282	FJ160833	FJ160878
Linum campanulatum		KJ194513			KJ194519	FJ169569	GQ845226	GQ845253	GQ845280	GQ845199	KJ194525
Linum strictum		FJ169530			FJ160806	FJ169590	GQ845235	GQ845262	GQ845289	FJ160845	FJ160890

Linum volkensii		FJ169531		HM544116	FJ160813	FJ169597					FJ160897
Linum setaceum		KJ194518			KJ194523						KJ194529
Linum tenuifolium		FJ169529		HE966947	FJ160809	FJ169594	GQ845236	GQ845263	GQ845290	FJ160848	FJ160893
Linum suffruticosum		FJ169532		HM544114	FJ160807	FJ169591				FJ160846	FJ160891
Linum trigynum		KU674815			FJ160810	FJ169595				FJ160849	FJ160894
Linum maritimum		KU674815			FJ160811	FJ169579				FJ160850	FJ160895
Linum tenue		FJ169548			FJ160808	FJ169593				FJ160847	FJ160892
Linum oligophyllum		FJ169546		HM544111	FJ160783	HM544072					FJ160867
Linum comptonii		FJ169550		HM544104	FJ160778	FJ169572				FJ160821	FJ160862
Linum sulcatum		MG235002				FJ169592					
Linum rupestre		FJ169553		HM544113	FJ160785	FJ169586				FJ160824	FJ160869
Linum rigidum var. *berlandieri*		MG236229		HM544101		FJ169567					
Linum kingii		FJ169555			FJ160780	FJ169576				FJ160823	FJ160864
Linum vernale		FJ169552			FJ160812					FJ160851	FJ160896
Sclerolinon digynum		FJ169541		AB233792	FJ160787	AB233896	KT453486			FJ160826	FJ160871
Hesperolinon micranthum		FJ169542		HM544086	FJ160775	FJ169561	KT453477			FJ160818	FJ011330
Cliococca selaginoides		KT453457		HM544080	FJ160774	FJ169558	KT453487				FJ160858
